# Systemic panca-associated vasculitis with central nervous involvement causing recurrent myelitis: case report

**DOI:** 10.1186/1471-2377-10-118

**Published:** 2010-11-30

**Authors:** Alexander J Hamilton, Duncan J Whitehead, Matthew D Bull, Richard J D'Souza

**Affiliations:** 1 Exeter Kidney Unit, Royal Devon and Exeter NHS Foundation Trust, Barrack Road, Exeter, Devon, EX2 5DW, UK

## Abstract

**Background:**

We report on the case of an established perinuclear antineutrophil cytoplasmic antibody (pANCA) associated renal vasculitis being treated with prednisolone and rituximab, where the patient presented with leg weakness, urinary and faecal incontinence and buttock pain consistent with transverse myelitis.

**Case presentation:**

The patient underwent MRI scanning showing patchy cord enhancement from T10 to the conus, which was suggestive of a cord malignancy. Prior to a cord biopsy, he was treated with steroids and a repeat MRI showed resolution of the original lesion with a new similar lesion from C7 to T3.

**Conclusions:**

He made a marked recovery after further treatment with high dose steroids and plasma exchange.

## Background

This case report highlights a possible presentation of spinal cord inflammation in which imaging initially suggested an alternative pathology to transverse myelitis, such as a spinal cord tumour. In addition it examines recurrent myelitis associated with a systemic pANCA associated vasculitis despite active immunosuppression, in particular the failure of rituximab and the requirement for plasma exchange.

There are a handful of reports of central nervous system involvement in patients with vasculitis. Many of these specifically involve Wegener's granulomatosis; López-Rodríguez et al[1] report on a case of Wegener's with a granulomatous mass in the Meckel cavum and diffuse meningeal involvement, treated with steroids and cyclophosphamide. Levin et al[2] report on a case of Wegener's granulomatosis with dural inflammation managed with steroids and cyclophosphamide. Their case highlights periaortitis as a particular feature. Nagashima et al[3] report on a case of pANCA Wegener's with hypertrophic pachymeningitis and multiple cranial neuropathies, this case similarly presented with an initial diagnosis suggesting malignancy that was disproved at biopsy.

Morinaga et al[4] report on a case of pANCA positivity associated with cerebellar dural enhancement as well as temporal arteritis. This patient had multiple cranial neuropathies.

The imaging features initially supporting a diagnosis of a cord tumour have been documented in the literature by Rourke et al[5]. In this case the abnormal area was macroscopically normal and intraoperative specimens did not show neoplastic cells, and the patient recovered with cyclophosphamide and steroids.

Rituximab, the chimeric anti-CD20 antibody was used in the treatment of this patient. Lovric et al[6] reported on a case series using rituximab as rescue therapy in ANCA positive vasculitis. Remission, whether partial or complete, was achieved in 14/15 patients. Rituximab has been used to treat neuromyelitis optica[7], and in our case although imaging features were consistent, there was no optic neuritis, a negative antibody to aquaporin-4 and poor response to rituximab.

Zandman-Goddard et al report on a patient with hepatitis C and persistent ANCA positivity[8]. Our patient tested negative for hepatitis C infection.

Our case highlights firstly the possibility of a serious central nervous system flare despite rituximab therapy, where the presentation imaging suggested an alternative pathology, which was successfully treated with an increased steroid dose and plasma exchange.

## Case presentation

A 65-year-old man presented in February 2007 with deteriorating renal function, creatinine 208 μmol/L (reference range 60-120 μmol/L). His background included chronic obstructive pulmonary disease and radiologically diagnosed asbestosis, previous duodenitis and pericarditis in 1970. His regular medications included a statin, a proton pump inhibitor, aspirin and an antimuscarinic bronchodilator.

His urinalysis showed protein 1+, blood 4+ and his serum immunology showed a positive pANCA and myeloperoxidase (MPO) antibody. His C-reactive protein (CRP) was 33 mg/L. He underwent renal biopsy which showed evidence of an inactive vasculitis; 25 glomeruli (11 normal, 7 sclerosed, 7 showing fibrous crescents, 0 showing cellular crescents). There was patchy tubular atrophy, focal secondary interstitial inflammation and fibrosis. Immunofluoresence was negative. Silver stain was unremarkable. This was all in keeping with a pANCA-associated renal vasculitis that was currently quiescent.

He maintained a stable creatinine with CRP <8 mg/L and was managed conservatively with close monitoring. In November 2007 it was noted he had a rising creatinine reaching 309 μmol/L prompting the initiation of prednisolone 80 mg and cyclophosphamide 200 mg with co-trimoxazole prophylaxis.

His creatinine fell to 146 μmol/L by April 2008 with this regimen and his prednisolone was slowly weaned. Cyclophosphamide was discontinued due to leucopaenia seven weeks after commencement and this was replaced with azathioprine 150 mg.

In July 2008 he had lower respiratory tract symptoms with raised inflammatory markers and was suspected of having active pulmonary involvement of his vasculitis. He underwent high resolution CT scanning of the chest which demonstrated areas of sub-pleural reticular shadowing, many related to areas of pleural thickening. He was noted to have a marked reduction in gas transfer (to 30% predicted), as well as a restrictive defect on spirometry. His azathioprine was changed to mycophenolate mofetil (MMF) 1 g bd as his CRP remained elevated (at 24 mg/L) with continued clinical vasculitic activity.

By September 2008 his creatinine rose to 198 μmol/L with continued pANCA positivity and his CRP was elevated at 60 mg/L. In view of cyclophosphamide induced leucopaenia and failure of control with MMF, he was commenced on Rituximab 375 mg/m^2 ^weekly for four weeks. At this stage his prednisolone dose was 25 mg.

**Figure 1:**
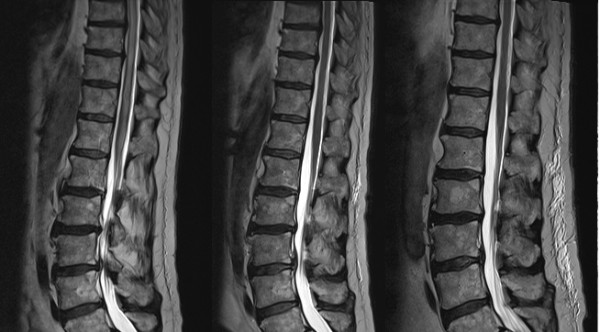
**Chronological MRI images of lumbar spine - November 2008/December 2008/May 2009**. In figure 1 the lumbar spine is most clearly affected in the first image (November 2008). The images show increased signal within the substance of the spinal cord during episodes of active vasculitis, with some subtle expansion of the cord.

**Figure 2:**
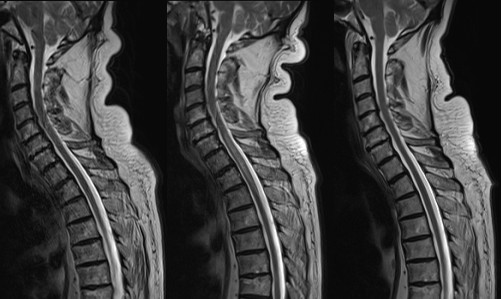
**Chronological MRI images of thoracic spine - November 2008/December 2008/May 2009**. In figure 2 the thoracic spine is most clearly affected in the second image (December 2008). The images show increased signal within the substance of the spinal cord during episodes of active vasculitis, with some subtle expansion of the cord.

In November 2008 he presented to his general practitioner with a three-week history of progressive weakness of the left leg and sensory loss up to T10 dermatome level followed by urinary and faecal retention. He complained of buttock pain and also some similar right-sided symptoms, which was consistent with a clinical diagnosis of transverse myelitis. There was no optic involvement. He was referred to the Spinal team who requested an MRI (Figures 1 &2) demonstrating patchy, slightly nodular central enhancement at T10 extending to the conus. MRI head was normal. The radiological differential diagnosis included a primary cord neoplasm such as astrocytoma or less likely ependymoma.

He was referred to the Neurologists and underwent CT scanning of the chest, abdomen and pelvis. This confirmed no primary malignancy or evidence of metastatic disease. He underwent CSF examination showing 10 × 10^6^/l lymphocytes with no malignant cells, protein of 0.9 g/L. An antibody to aquaporin-4 was negative, as was lupus and antiphospholipid serology.

He was referred for biopsy of the cord lesion. However, he had a boost in his dose of steroids and it was suggested to repeat the MRI scan before cord biopsy. The repeat MRI (Figures 1 &2) showed that the large lesion involving the lower thoracic cord had now almost completely resolved and the area of enhancement seen in the lower cord was no longer present. There was a new lesion extending from C7 down to T3. This was similar to the original lesion in that it was expansile and returned high signal on T2 weighted images with a much smaller area of central ring enhancement. It was felt that these changes made malignancy seem extremely unlikely as the affected areas had changed with time and increased immunosuppression. Vasculitis was suggested as a unifying diagnosis for his symptoms, the increase in steroids explaining the drastic MRI changes. His CRP peaked at 137 mg/L and dropped to 7 mg/L. Hence cord biopsy was not undertaken and his steroids continued at high dose, MMF stopped and rituximab continued. He was discharged and a further outpatient MRI showed continued improvement and his steroids were weaned down.

In May 2009 he returned to hospital following further left sided pain. He was treated for a vasculitis flare. He underwent repeat MRI head and spine. This showed a single high focus in the right cerebellar hemisphere. He was treated with intravenous methylprednisolone and further rituximab treatment was arranged. His last dose of rituximab was December 2009.

In February 2010 he was again admitted with left sided pain and a CRP of over 300 mg/L. There was no evidence of infection and his MRI was repeated which did not show any abnormal high signal on this occasion. His steroids were again increased. He was recommenced on MMF, which then had to be stopped owing to increased frequency of chest infections. He was noted to be Cushingoid as a result of prolonged steroid therapy.

In May 2010 he was commenced on plasma exchange which, after seven exchanges, successfully treated ongoing symptoms of leg pain attributed to spinal vasculitis and brought his CRP to below 3 mg/L. Plasma exchange is being continued at present due to continued pANCA positivity.

## Conclusions

The case presented highlights the difficulties in diagnosis, management and complications of vasculitis.

In the context of an established diagnosis of vasculitis, progression and extra-renal features were complicated by varying appearances on spinal MRI imaging, initially thought to be tumour. There were radical changes on subsequent images which led to questioning of the original diagnosis of spinal cord malignancy.

Finally the condition was resistant to rituximab but responded to high-dose steroids and then plasma exchange.

## Consent

Written informed consent was obtained from the patient for publication of this case report and any accompanying images. A copy of the written consent is available for review by the Editor-in-Chief of this journal.

## Competing interests

The authors declare that they have no competing interests.

We have had no involvements that might raise the question of bias in the work reported or in the conclusions, implications, or opinions stated.

## Authors' contributions

AH acquired the data via the case notes and drafted the manuscript. DW reviewed the literature and critically revised the drafted article. MB provided the images and legends. RD conceived the case report, critically revised the drafted article and gave final approval for publication. All authors read and approved the final manuscript.

## Author's information

AH is a Senior House Officer in Medicine. DW is a Registrar in Nephrology and General Internal Medicine. MB is a Registrar in Radiology. RD is a Consultant Nephrologist.

## Pre-publication history

The pre-publication history for this paper can be accessed here:

http://www.biomedcentral.com/1471-2377/10/118/prepub
